# Case report: Carbohydrate malabsorption in inpatients with anorexia nervosa

**DOI:** 10.3389/fpsyt.2022.1076658

**Published:** 2022-12-20

**Authors:** Patrizia Buck, Miriam Goebel-Stengel, Isabelle Mack, Stephan Zipfel, Andreas Stengel

**Affiliations:** ^1^Department of Psychosomatic Medicine and Psychotherapy, University Hospital Tübingen, Tübingen, Germany; ^2^Department of Internal Medicine, Helios Clinic, Rottweil, Germany; ^3^Department for Psychosomatic Medicine, Charité Center for Internal Medicine and Dermatology, Charité-Universitätsmedizin Berlin, Corporate Member of Freie Universität Berlin, Berlin Institute of Health, Humboldt-Universität zu Berlin, Berlin, Germany

**Keywords:** bloating, complaint, diarrhea, fructose malabsorption, eating disorder, gastrointestinal, hydrogen breath test, lactose intolerance

## Abstract

**Background:**

Gastrointestinal (GI) complaints are frequently observed in patients who suffer from anorexia nervosa (AN). These symptoms may hamper treatment and weight regain and are often perceived as the cause, not the consequence, of the disease. Since carbohydrate malabsorption also produces these symptoms, this might underly or contribute to these complaints. So far, the role of carbohydrate malabsorption (fructose malabsorption and lactose intolerance) in AN has not yet been investigated.

**Methods:**

For this case series, inpatients with AN of restrictive type (*n* = 3), purging type (*n* = 3), and atypical AN (*n* = 1) conducted hydrogen breath tests with 25 g of fructose and 50 g of lactose to investigate carbohydrate malabsorption. Results were then analyzed in association with body mass index (BMI) and patient-reported outcomes (disordered eating, body image disturbances, anxiety, depressive symptoms, perceived stress, and GI complaints).

**Results:**

Based on the hydrogen breath test results, three of the seven female patients were classified as lactose intolerant and one presented fructose malabsorption. Both hydrogen curves for fructose (*r* = –0.632, *p* < 0.001) and lactose (*r* = –0.704, *p* < 0.001) showed a negative correlation with BMI. No association was observed between hydrogen values and patient-reported outcomes.

**Conclusion:**

In patients with AN, GI symptoms caused by intolerance of common monosaccharides and disaccharides may be an underestimated burden and should be considered in the diagnosis and therapy of patients with AN. Due to the observed correlation with BMI, GI complaints after ingestion of fructose or lactose likely develop with decreasing body weight and are potentially reversible with weight regain.

## Introduction

Anorexia nervosa (AN) is a serious mental illness affecting the entire body with limited physical performance and impaired cognitive functions due to malnutrition (1). Although AN may be observed in middle-aged individuals and men, this disorder particularly affects female adolescents and young adult women (2). Characteristics of this eating disorder (ED) are defined in the Diagnostic and Statistical Manual of Mental Disorders 5 (DSM-5) and encompass “significantly low body weight” as a “core feature” due to “restriction of energy intake,” an intense “fear of gaining weight” and “disturbance in the way in which one’s body weight or shape is percepted and experienced.” Additionally, “persistent behavior that interferes with weight gain, even though at a significantly low weight,” promotes weight loss such as excessive training sessions or, depending on the existing subtype–restricting or binge-eating/purging–regular “self-induced vomiting or the misuse of laxatives diuretics or enemas” are diagnostic criteria for AN. The aforementioned specifications of AN are coded in the DSM-5 (3) and similarly (although not identically) listed in the ICD-10-CM (4). In addition, several psychological comorbidities (depression, anxiety disorders, and compulsivity) (1, 3) and somatic complications (e.g., fatigue, dizziness, osteoporosis, or constipation) in several organ systems are known (2).

More than 90% of the patients with AN report the presence of gastrointestinal (GI) complaints, such as postprandial fullness (5), bloating, abdominal pain, or diarrhea (6, 7). Furthermore, reduced intestinal motility and/or constipation are frequently reported (8). It is noted that patients with AN indicate the GI disturbances as the cause of the disturbed eating and not as their consequence (9).

The aforementioned symptoms are similar to those reported by subjects who are afflicted by malabsorption of common carbohydrates, such as fructose and lactose (10). These non-absorbed sugars reach the colon where they are fermented by colonic bacteria to gases including H_2_ (11). Malabsorption of fructose can be explained by reduced expression of the GLUT5 and GLUT2 transporters (12, 13), whereas the reason for impaired absorption of lactose is the enzymatic deficiency of lactase based on different conditions: due to congenital causes, acquired continuous decrease in enzyme activity, or developmental lactase deficiency, which altogether are described as primary lactose intolerance (14). The secondary hypolactasia results from an impaired small intestinal epithelium due to GI pathologies, such as celiac disease (14, 15) or external influences. For instance, lactose intolerance, which can be attributed to transient gut barrier dysfunction was found in patients receiving chemotherapy (16) or following viral gastroenteritis (17). Also, body weight loss might contribute to the development of carbohydrate malabsorption (18).

Studies examining a possible correlation between AN and the presence of malabsorption of specific monosaccharides and disaccharides are still missing (18, 19). One study merely examined the metabolization of glucose with a glucose tolerance test (20) which, however, indicates small intestinal bacterial overgrowth and not lactose intolerance or fructose malabsorption. The lactose and fructose hydrogen (H_2_) breath tests are the gold standard, which measures H_2_ content in an end-expiratory breath sample after oral ingestion of fructose or lactose. This can be used as an indicator of malabsorption if a significant H_2_ increase occurs along with typical GI symptoms (21). The aim of our study was therefore to investigate a case series of patients hospitalized for the treatment of their AN regarding the malabsorption of fructose and lactose.

## Materials and methods

### Study participants

The inclusion criteria for participation in the study were the diagnosis of AN based on the ICD-10 (F50.0 or F50.1) (4) and the hospitalization for the treatment of the AN. This group consisted of subjects with a restrictive type (F50.00, *n* = 3, patients 1, 4, and 6), a purging type (F50.01, *n* = 3, patients 2, 3, and 5), and atypical AN (F50.1, *n* = 1, patient 7). Patients were excluded if they had an oncological disease, psychotic disorder, existing pregnancy, or they were breastfeeding, and in the case of illegal drug use, medication intake in the last 3 months within a clinical trial (other medication–prescribed and over the counter–is indicated in Table 1) and disease (e.g., exocrine pancreatic insufficiency) or surgical procedure on the digestive tract or hereditary fructose intolerance.

**TABLE 1:** Diagnoses and current medication of inpatients.

Patient no.	Diagnoses	Current medication
1	AN restrictive type, moderate depressive episode	None
2	AN purging type	None
3	AN purging type, recurrent depressive disorder, current severe episode, acne papulopustulosa	Clindamycin 10 mg/g and tretinoin 0.25 mg/g gel
4	AN restrictive type	Estradiol 2 mg
5	AN purging type, severe depressive episode, post-traumatic stress disorder, social phobia, combined personality disorder with emotionally unstable, self-confident and anxious-avoidant components	Quetiapine 25 mg
6	AN restrictive type, recurrent depressive disorder, current severe episode, hypothyroidism	L-thyroxine 50 μg
7	Atypical AN, bronchial asthma	Montelukast 10 mg, ambroxol 30 mg, beclometason/formoterol 100/6 μg inhaler

Inpatients (aged 18 years and older) hospitalized in the University Hospital Tübingen, Department for Psychosomatic Medicine and Psychotherapy, were asked at admission to participate in the study with H_2_ breath testing during the first 3 days after admission and within the last week before discharge. No antibiotics or other drugs that may modify the gut microbiota composition had been administered 7 days before the H_2_ breath test. All patients provided written informed consent. The study was approved by the Local Ethics Committee (722/2018BO2).

### Patient-reported outcomes

During the first 3 days after admission and (originally intended) within the last week before discharge, patients electronically received validated questionnaires to assess eating behavior, (Eating Disorder Examination Questionnaire (EDE-Q) (22) and Eating Disorder Inventory-2 (EDI-2) (23); EDE-Q: Cronbach’s alpha for the current sample was 0.937; EDI-2: Cronbach’s alpha for the current sample was 0.908), body image (Fragebogen zum Körperbild-20, FKB-20–subdivided into rejecting body image and vital body dynamic (24), FKB-20, body image: Cronbach’s alpha for the current sample was 0.877; FKB-20, body dynamic: Cronbach’s alpha for the current sample was 0.812), anxiety (Generalized Anxiety Disorder Scale-7 (GAD-7) (25), Cronbach’s alpha for the current sample was 0.684), depressive symptoms (Patient Health Questionnaire-9 (PHQ-9) (26), Cronbach’s alpha for the current sample was 0.882), perceived stress (Perceived Stress Questionnaire-20 (PSQ-20) (27), Cronbach’s alpha for the current sample was 0.550), and GI symptoms (Gastrointestinal Symptom Rating Scale (GSRS) (28), Cronbach’s alpha for the current sample was 0.696). The total scores were used for the evaluation of all questionnaires. Only in the case of the GSRS, the subscales and an AN-specific total score (consisting of the GSRS subscales, which—as the study by Riedlinger et al. (7) showed—are most frequently pathological in patients with AN at the beginning of therapy: abdominal pain, indigestion, and constipation) were additionally calculated.

### H_2_ breath testing

The hydrogen breath tests for fructose (25 g) and lactose (50 g) were performed as described earlier (10). Overnight (at least 12 h)-fasted patients (only water but no consumption of nicotine or caffeine was permitted) underwent the test the following morning at 8 a.m. On the day before the H_2_ test, the subjects were advised not to eat any foods containing fiber, such as whole grains, beans, or lentils. They should also refrain from intense physical activity before and during the performance of the test. Likewise, intestinal stimulants or antibiotics had to be avoided the week before the hydrogen breath test due to the possible influence on the intestinal microbiota. Sample collection of end-expiratory exhaled air after oral carbohydrate ingestion was performed *via* a mouthpiece. At first, an H_2_ baseline value at exhalation was noted, and after this, all subjects received a solution consisting of 300 ml water and the defined amount of the respective carbohydrate (25 g fructose and 50 g lactose) as detailed in a position paper (21). After consumption of the solution (within 5 min), patients recorded and documented H_2_ concentration in the exhalation air at 15, 30, 60, 90, 120, 150, and 180 min. Reading of these results and documentation was done by the inpatients and no supervision was required by the protocol. At the same time, abdominal complaints were documented. H_2_ exhalation was assessed using the portable H_2_ sensor (Gastrolyzer, Bedfont, SpecialMed, Herrsching, Germany; measurement range 0–500 parts per million [ppm], cross-sensitivity <1%, measurement accuracy ± 2%) as performed earlier (10). Hydrogen values starting at ≤ 20 ppm and rising more than 20 ppm above the baseline were determined as pathological. A distinction must be made between the sole increase in H_2_ and a rising H_2_ value with simultaneous occurrence of known GI symptoms such as flatulence, diarrhea, nausea, or a feeling of fullness, with varying frequency and intensity as an indication of intolerance (21).

On the day of inpatient admission (and repeatedly as part of the clinical routine), blood was taken to assess blood count, electrolytes, and liver enzymes. In addition, elastase was determined once in the stool at the beginning of the therapy to exclude exocrine pancreatic insufficiency. Body weight was measured in patients wearing light underwear to calculate the BMI.

### Treatment

All participants received the same multimodal psychosomatic therapy, which encompassed individual and group therapy, nutrition counseling, supervised daily eating, joint cooking sessions, body and art therapy, relaxation techniques, and physical therapy. An accompanying drug therapy against tension and depression was indicated for patient 5 (quetiapine 25 mg) and has already been taken by the patient since 2019. Although psychotropics may alter gut microbiota diversity, no data exist so far for low-dose quetiapine (29).

### Statistical analyses

All data (BMI, laboratory results, H_2_ values, and patient-reported outcomes) were completely assessed at admission. However, against our initial plan, we were able to conduct H_2_ breath testing only in two patients at discharge (both H_2_ tests in patient 1 and one H_2_ test in patient 6). The other patients either denied the conductance of the H_2_ tests at discharge (patients 2, 4, 6, and 7), were discharged prematurely (patient 3), or were transferred to the psychiatry ward due to increasingly diminished emotional regulation capacity with escalating self-injurious behavior over the course of the inpatient treatment (patient 5). Therefore, we were not able to longitudinally assess these data and report only the data from admission in this case series.

For statistical analyses, the program SPSS version 28.0.1.1 (IBM) was used. Collected data were analyzed descriptively and each item (sociodemographic/somatic data, laboratory results, H_2_ values, and patient-reported outcomes) was checked for normality using the Shapiro–Wilk test and visually with the QQ plot. Correlations were assessed using Spearman’s rank correlation.

## Results

Diagnosed according to standard face-to-face interviews using the national German S3 guidelines for the assessment and therapy of eating disorders (EDs) (30), seven female subjects between 18 and 42 years of age (mean: 30.0 years) and of German origin (Caucasian) were included in the group of test subjects. All of them were admitted as inpatients due to their non-improving ED, and among them, three were with a BMI of <15 mg/m^2^ (BMI range: 14.3–15.8 15 mg/m^2^, mean value: 15.1 15 mg/m^2^). On admission, all inpatients were asked about their physical and mental health over the past 2 weeks (response options ranged from not impaired to severely impaired) and rated their status in both health domains (Table 2). Regarding physical condition, the ED influenced the inpatients’ personal efficiency in their everyday activities (education/work and household), since all of them indicated a medium personal performance level (scale value of 4 or 5 on a Likert scale of 0–10). These scores showed that all female patients were still able to perform their daily tasks and nearly half of the patients felt unable to work. The blood values obtained at the same time did not show any abnormalities (Table 2), and stool elastase was normal in all subjects (> 500 μg/g). Six of the seven inpatients were already undergoing psychotherapeutic treatment at the time of the initial interview.

**TABLE 2:** Descriptive analysis of anthropometric, sociodemographic, laboratory, and psychometric data.

**Parameter**	Mean (±SD, Range), Median [Q1, Q3, IQR], {%}*
**Anthropometric data**	
Age	30.00 (8.51, 18–42)
Body weight	40.16 (3.2, 35.7–45.6)
Height	1.63 (0.04, 1.58–1.70)
BMI	15.09 (0.66, 14.30–15.78)
**Sociodemographic and somatic data**	
Impairment of physical health: mildly, moderately, severely	{28.6}, {57.1}, {14.3}
Impairment of mental health: mildly, moderately, severely	{28.6}, {28.6}, {42.9}
Nationality: German	{100}
Marital status: single, married	{85.7}, {14.3}
Living situation: alone, with partner/parents/family	{42.9}, {57.2}
Sex: female	{100}
Graduation: school-leaving qualification, completed degree	{85.8}, {14.3}
Professional status: Employed, studies, voluntary year	{71.4}, {14.3}, {14.3}
Inability to work: yes, no	{57.1}, {42.9}
Retired: no	{100}
Days of inactivity due to disease (last 2 weeks): yes, no	{28.6}, {71.4}
Medication (sedative, sleep, anti-depressants): no	{100}
Psychotherapy: yes (under treatment), no (never)	{85.7}, {14.3}
Smoker: yes, no	{28.6}, {71.4}
Days of hospitalization (last 3 months): yes, no	{28.6}, {71.4}
Requested treatment program: inpatient treatment, day-care hospital, others	{46.2}, {23.1}, {30.8}
Number of doctor’s visits (last 4 weeks)	2 [1, 2, 1]
Personal performance^a^ (last 2 weeks)	5 [4, 5, 1]
Intensity of pain^a^ (last 2 weeks)	1 [0, 2, 2]
Intensity of depressions^a^ (last 2 weeks)	2 [0, 2, 2]
Intensity of fears^a^ (last 2 weeks)	0 [0, 3, 3]
**Lab results**	
Leukocytes [per μl]	4,890 (3,066, 1,500–11,070)
Erythrocytes [million/μl]	4.27 (0.46, 3.43–4.74)
Hb [g/dl]	13.43 (1.33, 12.0–15.6)
HCT [%]	37.44 (3.46, 32.6–42.5)
MCV [fl]	87.9 (5.39, 81.1–95.0)
MCH [pg]	31.54 (2.58, 27.3–35.0)
MCHC[g/dl]	35.86 (1.04, 33.7–36.8)
Thrombocytes [1,000/μl]	250 (68, 187–406)
GOT/AST [U/l]	21.86 (17.90, 6–59)
GPT/ALT [U/l]	29.57 (33.96, 11–106)
Urea [mg/dl]	27.29 (14.58, 14–53)
Electrolytes [mmol/l]	Na^+^: 138.86 (5.11, 129–146), K^+^: 3.99 (0.76, 2.9–5.3) Ca^2+^: 2.31 (0.12, 2.2–2.5), PO_4_: 1.19 (0.13, 0.9–1.3)
Total protein [g/dl]	7.17 (0.45, 6.3–7.6)
**Psychometric data**	
EDE-Q	3.28 (1.83, 1.27–5.19)
EDI-2 (scaled to 100)	53.85 (13.14, 40.84–74.36)
FKB-20 (body dynamic)	25.14 (7.99, 14–37)
FKB-20 (body image)	35.71 (12.78, 21–50)
GAD-7	7.14 (4.10, 0–12)
PHQ-9	11.29 (6.26, 2–18)
PSQ-20	53.57 (22.47, 18.33–85)
GSRS total, GSRS AN-specific, abdominal pain, reflux, diarrhea, indigestion, constipation	3.16 (0.77, 1.80–3.93), 3.52 (1.27, 1.94–5.00), 2.91 (1.24, 1.67–4.67), 1.93 (1.06, 1.00–4.00), 2.48 (1.80, 1.00–5.67) 4.46 (1.50, 2.50-6.25), 3.19 (1.72, 1.00-5.33)

*At the beginning of inpatient therapy, *n* = 7; ^a^On a scale from 0 to 10 (0 = non-existent, 10 = greatest possible).

After the consumption of 25 g fructose, only one patient (patient 3) reported several GI complaints (flatulence, bloating, stomach pains, and diarrhea) in addition to a pronounced (> 20 ppm) rise in breath H_2_ levels (H_2_ value range: 2–26), indicative of fructose malabsorption. All other patients were not classified as fructose malabsorbers, and the reported GI symptoms were stomach pain in patient 1 after 60 min (H_2_ value range: 0–4). In patient 2 directly after fructose ingestion, mild stomach pain was reported (H_2_ value range: 0–3), patient 4 complained about mild nausea after 90 min (H_2_ value range: 2–5), patients 5 and 6 reported no GI symptoms, and patient 7 already showed a higher H_2_ value at the beginning of the fructose test but only increased slightly (H_2_ value range: 21–36), with the simultaneous existence of only mild stomach pain after 15 min.

After intake of 50 g of lactose, three of the seven patients reported GI and other complaints (flatulence, bloating, stomach pain, diarrhea, nausea, borborygmi, headaches, and discomfort) associated with a significant rise in H_2_ levels. Therefore, three out of the seven patients (patients 3, 6, and 7) were classified as lactose intolerant. In the other non-lactose intolerant patients, neither rising H_2_ values nor corresponding GI symptoms were observed: patient 1 reported stomach pain and hunger from the 60th min (H_2_ value range: 0–2), patient 2 had mild stomach pain after 15 min (H_2_ value range: 0–2), patient 4 mentioned mild nausea (H_2_ value range: 2–6), and patient 5 noticed mild rumbling in the stomach at 30 and 90 min (H_2_ value range: 0–1).

With regard to the established lactose intolerance in patient 3, she reported that she did not tolerate lactose well at low body weights. This observation was made during the eating disorder (diagnosed more than 4 years ago) with the lowest weight of 36 kg (BMI: 14.2). She reported tolerating lactose at higher body weights. The other two inpatients who reacted strongly to lactose intake (patients 6 and 7) had a long history of AN (10–20 years) as well.

GI symptoms due to other causes such as hepatic or pancreatic dysfunction could be excluded for all participants based on the blood test results (Table 2). Only one patient (patient 1) showed slightly elevated levels for GOT and GPT indicating liver damage as a possible consequence of renutrition after a long period of malnutrition.

**FIGURE 1:**
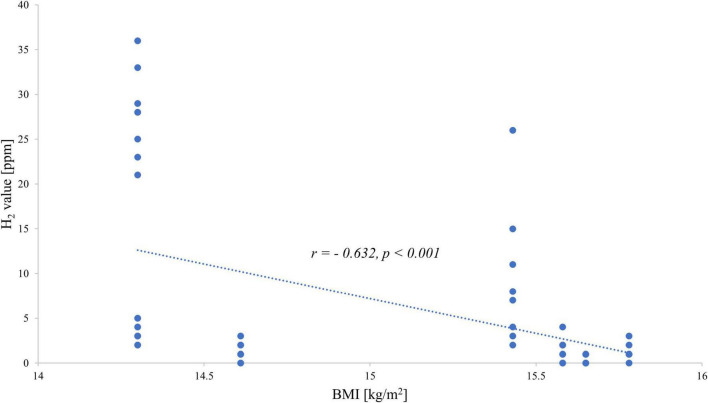
Correlation between end-expiratory hydrogen values during the fructose breath test and body mass index.

**FIGURE 2:**
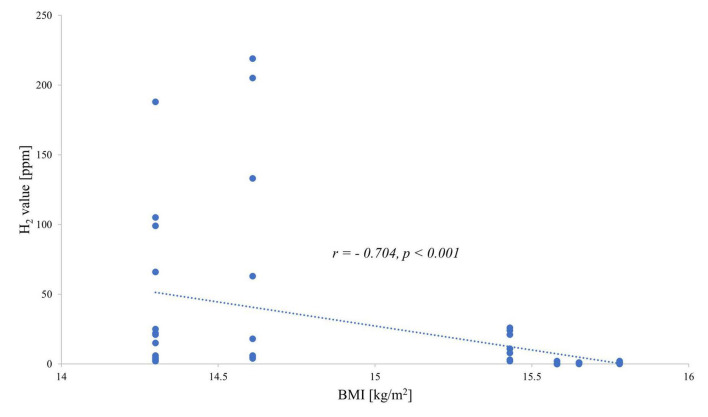
Correlation between end-expiratory hydrogen values during the lactose breath test and body mass index.

When investigating the exhaled H_2_ values and the BMI of each patient, a negative correlation was observed: the hydrogen value increased with decreasing BMI for both fructose (Figure 1) and lactose (Figure 2).

Patient-reported outcomes using validated questionnaires at admission are shown in Table 2. The severity level of ED reflected in the EDE-Q (22) was below levels compared with previous studies on patients with EDs (22, 31) with scores of 4 or higher (current mean EDE-Q score: 3.28). The assessment of attitudinal and behavioral features corresponding to AN or bulimia nervosa by EDI-2 (23) was higher than levels reported before in patients with AN (7, 32) (mean EDI-2 score scaled to 100: 53.9). For the assessment of body image, both subscales of the questionnaire FKB-20 were considered. The conception of the energetic aspect was similar to values previously recorded for patients with AN or depressive disorder but below the value calculated for patients with a somatoform disorder (33) (mean FKB-20 body dynamics score: 25.1). The body image score (mean: 35.7) was higher in comparison to the one observed in the mentioned anorexic group and patients with a somatoform disorder (33). Anxiety was reported as mild (mean GAD-7 score: 7.1) (25), and therefore higher compared with the general population (34) but lower than previously reported in patients with AN (7). Depressive symptoms (PHQ-9, score 0–4: minimal, score 5–9: mild, score 10–14: moderate, score 15–19: moderately severe, scale 20–27: severe) (35) were reported to be moderate in the current sample (mean PHQ-9 score: 11.3) consistent with a previous study on patients with AN (7). The perception of stress assessed using the PSQ-20 was above levels reported before for healthy adults and similar to the levels described in psychosomatic outpatients (27) (current mean PSQ-20 score: 53.6).

Frequency and intensity of GI complaints assessed by the GSRS showed increased values compared with previous studies indicating a value of ≥2 in GSRS scores as pathological (36, 37) (current mean GSRS total score: 3.2). The GSRS is composed of five subscales representing GI symptom clusters, such as abdominal pain, constipation, diarrhea, indigestion, and reflux (7). Reported symptoms of the seven female inpatients were indigestion (mean: 4.5) and constipation (mean: 3.2), followed by abdominal pain (mean: 2.9), diarrhea (mean: 2.5), and reflux (mean: 1.9). The first three symptoms are the most prevalent in AN (7). The AN-specific GI symptom scale (mean GSRS AN-typical score: 3.5) was comparable to the results of the study by Riedlinger et al. (7) assessing the prevalence of GI complaints in patients with AN during admission and discharge of therapy. The mean GSRS total score compared with non-psychiatric controls was higher but lower than the levels observed in outpatients with obsessive-compulsive disorder with and without irritable bowel syndrome (38). Comparison of each GSRS subscale with those of patients with functional dyspepsia showed similar levels (39). No associations were observed between exhaled H_2_ levels and any patient-reported outcome (Table 3).

**TABLE 3:** Correlation between patient-reported outcomes of inpatients and AUC of H_2_ values after provocation with fructose or lactose.

Psychometrictest	Fructose		Lactose	
	*r*	*P*	*r*	*p*
*EDE*−*Q*	-0.607	0.148	-0.679	0.094
*EDI*−2	-0.505	0.248	-0.180	0.699
*FKB*−20(*bodydynamic*)	-0.286	0.535	0.179	0.702
*FKB*−20(*bodyimage*)	-0.054	0.908	-0.270	0.558
*GAD*−7	-0.569	0.182	-0.220	0.635
*PHQ*−9	-0.342	0.452	-0.306	0.504
*PSQ*−20	-0.071	0.879	-0.464	0.294
*GSRStotal*	0.071	0.879	0.214	0.645

## Discussion

In the present study, we conducted hydrogen breath tests with fructose and lactose in female inpatients with AN to examine whether the malabsorption of these carbohydrates might be responsible for the frequently reported GI symptoms. Although malabsorption *per se* was not particularly frequent in our current sample, both H_2_ values after fructose and lactose showed a close inverse correlation with BMI giving rise to the possible development of fructose malabsorption and lactose intolerance as a consequence of decreasing body weight. The study of Friesen et al. (19) tested the occurrence of malabsorption after ingestion of a solution made of 25 g fructose (and 5 g sorbitol) in 26 female patients with eating disorders and a BMI of 18.6 ± 3.6 kg/m^2^; among them, 10 were with AN, in comparison to normal weight female controls. Over a testing period of 3 h, GI complaints were hourly noted in addition to the conduction of a hydrogen breath test: eight inpatients with anorexia reported more frequent and severe GI symptoms (abdominal pain, bloating, nausea, or flatulence) after fructose(-sorbitol) ingestion and 13 of the 26 patients with ED malabsorption was detected based on the hydrogen breath levels. Interestingly, they also indicated greater GI symptom responses in patients with a lower BMI, and therefore more common in patients with AN (19).

The likelihood of developing an intolerance at low body weight or restricted eating behavior also increased for lactose as shown in the study of Täljemark et al. (40). Therein, 12 of 95 children with restrictive eating problems, including three patients with AN, noticed lactose intolerance as one of the most prevalent coexisting GI problems. Low body weight due to severe weight loss in the context of cachexia has also been mentioned earlier as a possible cause of malabsorption entailed by skeletal muscle wasting, leading to the interaction with other tissues/organ systems, including the gut (41). A so-called gut barrier dysfunction might occur which may be responsible for/contribute to malabsorption (42). Another previous study showed that the GI microbiota was perturbed in AN when compared with normal-weight participants reflected by the decreased microbial richness and a shift in bacteria abundance: pro-inflammatory and mucin-degrading bacteria were increased, whereas a decrease of intestinal protective species and carbohydrate-utilizing taxa was observed (43, 44). Especially, *Roseburia* spp., being a key butyrate producer and important for gut health, appears to be decreased and positively correlated with BMI (45). After weight gain, the GI microbiota composition changes in AN. However, this composition is still distinct from that of healthy normal-weight participants (44). Based on these data, we hypothesize that malabsorption develops with decreasing BMI, which might hamper therapy but may normalize during weight restoration treatment of AN. To further confirm this hypothesis, a larger and longitudinal study is desired.

The assessment of patient-reported outcomes did not indicate an association between eating disorder symptoms and H_2_ values, further pointing toward malabsorption being most likely not the cause but the consequence of AN.

Patient 3 reacted positively to both fructose and lactose. A significant H_2_ increase of ≥ 20 ppm over the baseline was found in both the fructose and lactose breath tests after 90 min. Due to this combined H_2_ increase, small intestinal bacterial overgrowth (SIBO) and celiac disease should be excluded for this patient (10), which was suggested but not performed during her stay. Nonetheless, SIBO in this patient is not likely as the H_2_ increase in the performed tests was delayed.

Despite the strength of the study adding first data on the yet unexplored field of carbohydrate malabsorption in the context of AN, some limitations should be mentioned as well. First, the study population is very small, mostly because inpatients with severe AN often are unwilling—due to the avoidance of carbohydrates—or unable—due to the severity of the disease—to participate in this type of study. Second, the study was performed—against our initial plan—only cross-sectionally as patients were discharged early, transferred to another ward, or refused to participate in the H_2_ test at the time of discharge. The rate of discontinuation was 85.7% on performing the second fructose breath test and 71.4% on conducting the second lactose breath test. Third, other exclusion criteria might have been missed, which might confound the results. Finally, SIBO has not been ruled out *via* endoscopy and aspiration. Therefore, at this point, we can only speculate on the cause and consequence of carbohydrate malabsorption. For this purpose, a larger, possibly also including patients with less severe AN, and a longitudinal study with retesting after weight restoration is warranted to answer this question.

In this study, we showed that H_2_ levels after fructose and lactose challenges exhibit an inverse association with BMI, and therefore underweight might contribute/lead to reduced capacity of carbohydrate absorption in patients with AN, thereby hampering treatment. This possible cause–consequence relationship should be further investigated in longitudinal studies.

## Data availability statement

The raw data supporting the conclusions of this article will be made available by the authors, without undue reservation.

## Ethics statement

The studies involving human participants were reviewed and approved by Local Ethics Committee (722/2018BO2). The patients/participants provided their written informed consent to participate in this study.

## Author contributions

AS designed the study, conceived, and planned the experiments in collaboration with MG-S, IM, and SZ. IM supervised the clinical measurements. PB carried out the calculations and wrote the manuscript with input from all the authors.
